# Rapid assessment of regional SARS-CoV-2 community transmission through a convenience sample of healthcare workers, the Netherlands, March 2020

**DOI:** 10.2807/1560-7917.ES.2020.25.12.2000334

**Published:** 2020-03-26

**Authors:** Chantal B Reusken, Anton Buiting, Chantal Bleeker-Rovers, Bram Diederen, Mariëtte Hooiveld, Ingrid Friesema, Marion Koopmans, Titia Kortbeek, Suzanne PM Lutgens, Adam Meijer, Jean-Luc Murk, Ilse Overdevest, Thera Trienekens, Aura Timen, Wouter Van den Bijllaardt, Jaap Van Dissel, Arianne Van Gageldonk-Lafeber, Dewi Van der Vegt, Peter C Wever, Wim Van der Hoek, Jan Kluytmans

**Affiliations:** 1 Centre for Infectious Disease Control-National Institute for Public Health and the Environment, Bilthoven, the Netherlands; 2 These authors contributed equally to this work; 3 Elisabeth-Tweesteden hospital, Tilburg and Waalwijk, the Netherlands; 4 Radboudumc, Nijmegen, the Netherlands; 5 Bravis hospital, Roosendaal and Bergen-op-Zoom, the Netherlands.; 6 Nivel, Netherlands institute for health services research, Utrecht, the Netherlands; 7 Erasmus MC, Rotterdam, the Netherlands; 8 Jeroen Bosch Hospital, 's-Hertogenbosch, the Netherlands; 9 Catharina hospital, Eindhoven, the Netherlands; 10 VieCuri hospital, Venlo, the Netherlands; 11 Amphia hospital, Breda, the Netherlands; 12 Elkerliek hospital, Helmond, the Netherlands; 13 Bernhoven hospital, Uden, the Netherlands

**Keywords:** SARS-CoV-2, COVID-19, community transmission, healthcare worker, public health response, respiratory disease

## Abstract

To rapidly assess possible community transmission in Noord-Brabant, the Netherlands, healthcare workers (HCW) with mild respiratory complaints and without epidemiological link (contact with confirmed case or visited areas with active circulation) were tested for severe acute respiratory syndrome coronavirus 2 (SARS-CoV-2). Within 2 days, 1,097 HCW in nine hospitals were tested; 45 (4.1%) were positive. Of six hospitals with positive HCW, two accounted for 38 positive HCW. The results informed local and national risk management.

On 27 February 2020, the first case of coronavirus disease (COVID-19) was diagnosed in the Netherlands [1]. By 6 March, the number of cases had increased to 128 [2]. Most of these cases had a travel history to northern Italy or had been in close (household) contact with a laboratory-confirmed case. For 15 of the 128 cases the source of infection had not been determined. For seven of the 35 cases in the province of Noord-Brabant, the source of infection could not be established. Some cases elsewhere in the Netherlands were also linked to Noord-Brabant. Furthermore, in hospital B in Breda, which has offered low-threshold testing for employees with respiratory complaints since 2 March 2020, several healthcare workers (HCW) had tested positive for severe acute respiratory syndrome coronavirus 2 (SARS-CoV-2). On Friday 6 March, the Dutch National Outbreak Management Team (OMT) convened to discuss the situation of coronavirus disease (COVID-19) in the Netherlands. The OMT decided that an urgent assessment of possible community transmission in the province of Noord-Brabant was needed. 

## Sampling of healthcare workers for SARS-CoV-2

The OMT decided to approach the assessment of possible community transmission in Noord-Brabant through sampling of HCW in hospitals in the province. A focus on HCW would simplify sampling, at such short notice, of adequate numbers of people with mild respiratory symptoms (coughing and/or sore throat and/or common cold) and without a known epidemiological link for SARS-CoV-2 exposure (travel to high-risk areas, close contact with confirmed case). Furthermore, knowledge of the status of SARS-CoV-2 infection among HCW would provide important insights for the participating hospitals regarding the infection status of staff and would inform hospital policies on testing algorithms for their personnel and on infection prevention measures.

Seven hospitals in the province of Noord-Brabant were approached in the afternoon of Friday 6 March and the morning of Saturday 7 March 2020 with the request to test HCW through Sunday 8 March. Two hospitals indicated that they had already started systematic sampling of HCW as part of their hospital policy. Four hospitals had no systematic sampling policy for HCW but were testing all patients that presented at the emergency ward with respiratory complaints. In addition, two hospitals just outside Noord-Brabant with a large proportion of staff residing in the affected province participated in the assessment (Figure). The participating hospitals were asked to offer screening to HCW and share the results of the testing by 14:00 on Monday 9 March 2020. Upper respiratory tract specimens (throat and/or nasopharyngeal swab) were collected from HCW with mild respiratory complaints and without epidemiological link. Testing followed a uniform national protocol based on Corman et al. [3], that was rolled out by two central laboratories in the Netherlands. The testing was done either locally or in one of these two central laboratories. 

**Figure:**
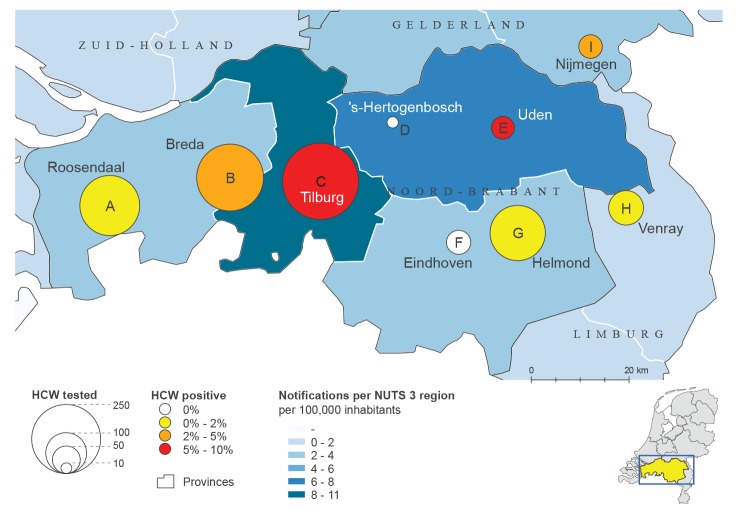
Locations of the nine participating hospitals, with numbers of healthcare workers tested for SARS-CoV-2 and percentage of positive test results, the Netherlands, March 2020 (n = 1,097 tests)

COVID-19: coronavirus disease; HCW: healthcare worker; NUTS: nomenclature of territorial units for statistics; SARS-CoV: severe acute respiratory syndrome coronavirus.

The background incidence of COVID-19 notifications by NUTS 3 region is based upon the national infectious diseases notification system.

Ethical approval was not required for this study as only anonymous aggregated data were used, and no medical interventions were made on human subjects. Sampling of HCW or patients was part of hospital policy.

In the period 6–8 March 2020, a total of 1,097 HCW (range per hospital: 11–294) in nine hospitals were tested for SARS-CoV-2, of whom 45 (4.1%) were found positive (Figure). Six hospitals had positive HCW of which two (hospital B and C) accounted for 38 of the 45 positive HCW. The percentage of positive HCW per hospital varied between 0% and 9.5% with the highest percentages in hospital B in Breda (4.2%; 10/238), hospital E in Uden (5.6%; 2/36) and hospital C in Tilburg (9.5%; 28/294). 

In addition, seven of the nine hospitals (A in Roosendaal, B in Breda, C in Tilburg, D in ‘s-Hertogenbosch, E in Uden, F in Eindhoven and I in Nijmegen) had already tested HCW in the period from 27 February to 6 March 2020. They reported 10 positive HCW among 400 tested (2.5%). The percentage of positive HCW per hospital varied between 0% and 5.6% in this period with the highest percentage in one hospital (D) in ‘s-Hertogenbosch.

In total, in the period from 27 February to 8 March 2020, four of the nine hospitals had tested 786 patients with respiratory complaints, of whom 27 (3.4%) were positive. The percentage of positive patients varied between 1.1% (1/87) and 16.2% (16/99), with the highest percentage in hospital E.

## Discussion

Since its first emergence in China in December 2019, SARS-CoV-2 has caused a pandemic affecting 134 countries with a total of 142,539 COVID-19 cases including 5,393 deaths by 14 March 2020 [4]. Fatal outcome was reported in the largest study from China to be 2,3% [5]. As at 15 March 2020, the Netherlands had officially registered 1,135 patients, with the majority of cases in the south-western part of the country [6]. Currently (15 March 2020), evidence is accumulating for unnoticed community transmission in the provinces Noord-Brabant and Limburg, with sporadic cases with unknown sources of infection elsewhere in the country.

A 2-day rapid study among nine hospitals with HCW working and/or residing in an area of the Netherlands with suspected community transmission showed that 4.1% of hospital staff with mild respiratory symptoms were infected with SARS-CoV-2. The observed geographic differences in positivity rates among HCW demonstrated focality of SARS-CoV-2 infection with foci in the region Breda–Tilburg and Uden. SARS-CoV-2 infections among patients with respiratory complaints were primarily found in the hospital in Uden. Source and contact tracing was started by the regional public health service upon positive testing in the patients.

The results of the rapid assessment confirmed the suspicions at the OMT meeting on 6 March 2020 that unnoticed community transmission was ongoing in parts of Noord-Brabant. The results directly informed decisions on control measures at the national level (9 March) and subsequently for additional regional measures (10 March). The study supported the implemented mitigation policy that was advised by the OMT on 6 March in anticipation of the results of the assessment [7]. The additional measures undertaken by regional authorities involved requesting inhabitants of Noord-Brabant to practice self-isolation at home when they developed a cough, symptoms of common cold and/or a fever. Furthermore, a ban of public events involving more than 1,000 people was implemented in this province [8]. As the epidemiological situation developed, on 12 March 2020, self-isolation upon mild respiratory symptoms was implemented for the whole country, together with a ban of events with more than 100 people [9]. Tailored advice was issued for people 70 years and older, persons belonging to medical risk groups and for persons involved with their care.

Here, we used SARS-CoV-2 infection rates among HCW with mild respiratory complaints without an epidemiological link as a proxy for community transmission. As the study had to be conducted under enormous time constraints (started and completed within 2 days) to be able to rapidly inform urgent decision making, there was no opportunity to roll out a standardised study protocol. Nevertheless, data provided by the World Health Organization-China joint mission on COVID-19, support our approach. The mission report indicated that there were 2,055 laboratory-confirmed cases of COVID-19 among HCW from 476 hospitals in China. Close investigation into these cases revealed that most of them could be traced back to exposure in households rather than in a healthcare setting [10].

We interpret the prevalence of 4% among HCW with mild respiratory illness and no epidemiological link as high and of concern. It suggests unnoticed community transmission, with a potential risk of nosocomial transmission. Further evidence for ongoing community transmission was provided by the Nivel Primary Care Database sentinel surveillance for influenza-like illness (ILI) and other acute respiratory infections (ARI) [11]. While this is a small group of ca 40 practices covering 0.8% of the Dutch population, eight ILI or ARI patients had tested positive by 14 March, one among 109 (0.9%) with a collection date in week 10 and nine among 125 (7.23%) in week 11. The epidemiological situation in the Netherlands and elsewhere is developing rapidly, and additional measures involving further restrictions in the social life in the country are being prepared.
